# Chromatin-modifying agents convert fibroblasts to OCT4+ and VEGFR-2+ capillary tube-forming cells

**DOI:** 10.1371/journal.pone.0176496

**Published:** 2017-05-03

**Authors:** Anita Wary, Neil Wary, Jugajyoti Baruah, Victoria Mastej, Kishore K. Wary

**Affiliations:** 1 York Community High School, Elmhurst, Illinois, United States of America; 2 Illinois Mathematics and Science Academy, Aurora, Illinois, United States of America; 3 Department of Pharmacology, University of Illinois at Chicago, Chicago, Illinois, United States of America; University of Texas at Austin Dell Medical School, UNITED STATES

## Abstract

**Rationale:**

The human epigenome is plastic. The goal of this study was to address if fibroblast cells can be epigenetically modified to promote neovessel formation.

**Methods and results:**

Here, we used highly abundant human adult dermal fibroblast cells (hADFCs) that were treated with the chromatin-modifying agents 5-aza-2'-deoxycytidine and trichostatin A, and subsequently subjected to differentiation by activating Wnt signaling. Our results show that these epigenetically modified hADFCs increasingly expressed β-catenin, pluripotency factor octamer-binding transcription factor-4 (OCT4, also known as POU5F1), and endothelial cell (EC) marker called vascular endothelial growth factor receptor-2 (VEGFR-2, also known as Fetal Liver Kinase-1). In microscopic analysis, β-catenin localized to cell-cell contact points, while OCT4 was found to be localized primarily to the nucleus of these cells. Furthermore, in a chromatin immunoprecipitation experiment, OCT4 bound to the *VEGFR-2/FLK1* promoter. Finally, these modified hADFCs also transduced Wnt signaling. Importantly, on a two-dimensional (2D) gel substrate, a subset of the converted cells formed vascular network-like structures in the presence of VEGF.

**Conclusion:**

Chromatin-modifying agents converted hADFCs to OCT4+ and VEGFR-2+ capillary tube-forming cells in a 2D matrix in VEGF-dependent manner.

## Introduction

Myocardial ischemia and cardiovascular diseases (CVD) are among the most significant causes of death and disability in the United States [**1–3**]. Despite the progress made in understanding the physiology of blood vessels and the heart, there are no effective therapies to fight many CVDs [**4–10**]. The use endothelial progenitor cells (EPCs) and induced pluripotent stem (iPS) cells to re-establish blood flow in ischemic tissues have met with limited clinical success [**11–16**]. Moreover, functional EPCs are not abundantly present in patients with CVDs [**17,18**]. Therefore, alternative sources and effective methods are required to generate replacement cells to promote new blood vessel formation.

Endothelial cells (ECs) lining the blood vessel walls not only play a significant role in regulating the flow of nutrients and oxygen, but also form an atheroprotective and anti-thrombotic surface [**19,20**]. Mature ECs are arrested at the G_0_-phase of the cell cycle and can only undergo 6 to 7 rounds of mitosis [**19–22**], and therefore do not divide at an adequate rate to replace the damaged and dying ECs. After EC damage and dysfunction, several cell types take part in the repair process [**23–25**], such as immune cells, fibroblasts, and possibly EPCs (**20**). The repair process can be normal or abnormal, with abnormal repair characterized by the presence of a scar [**25, 26**]. Experiments in animals show that the blood vessels formed as a consequence of wound healing harbor fibroblasts and macrophages [**27–29**]. Fibroblasts usually remain dormant, but are frequently found to be activated within newly formed blood vessels [**20**,**29–32**], or become activated in response to injury. Some observations suggest that fibroblasts can transition into an immature phenotype, often called myofibroblasts [**31,33**]. Recent studies showed that only a handful of genes are necessary to reprogram somatic cells; retroviral-mediated forced expression of transcription factors (TFs) Sox2, Oct4, KLF4, and c-Myc converted mature fibroblasts to iPS cells [**33–36**]. These TFs may bind directly or indirectly to the promoter or enhancer elements of specific genes, which in turn induce gene expression, thereby modulating the function of the cells. The gene expression induced by TFs could be high or low, constitutive or transient, and could be developmentally regulated. However, when stem cells differentiate into specific lineages *in vivo*, most of these embryonic genes, including *Sox2*, *Oct4*, *KLF4*, and *c-Myc*, are no longer expressed at high levels, or are silenced by epigenetic modification [**37–39**]. These epigenetic changes, such as methylation and acetylation in specific segments of promoters/enhancers, are responsible for maintaining a fully differentiated cell state [**37–39**]. For these reasons, ECs, cardiomyocytes, and smooth muscle cells (SMCs) fail to enter the cell cycle rapidly in response to injury or ischemia.

The formation of a primary vascular network, called vasculogenesis, requires a series of complex biological events that are regulated in time and space [**20,40–44**]. Studies have revealed the list of genes required to give rise to or maintain the EC phenotype, including: *Brachyury*, *Chloche*, *Er71/Etv2*, *FoxC2*, *VEGFR-2/Flk1*, *VE-cadherin*, *Hes1*, *Hey2*, *Notch-1*, *Tie-2*, and *LPP3* [**20,40–46**]. These genes are likely epigenetically modified or silenced in somatic cells [**47,48**]. Histone deacetylases (HDACs) are enzymes that remove acetyl groups from lysine residues on the N-terminal tails of histones [**47,48**]. Trichostatin A (TSA), an HDAC inhibitor, can increase acetylation of histones, leading to a less compact and thus more transcriptionally active chromatin [**47–51**]. The DNA methyl transferase (DNMT) inhibitor 5-Aza-2'-deoxycytidine (Aza) reduces methylation, which leads to increased activation of the associated genes [**47–51**]. Tideglusib (TDG) is an allosteric inhibitor of glycogen synthase kinase-3β (GSK-3β); inhibition of GSK-3β prevents phosphorylation of its substrate β-catenin [**52–54**]. The lack of phosphorylation stabilizes β-catenin, which then translocates to the nucleus, where it activates expression of Wnt target genes that control cellular fate and differentiation.

Here, we focus on fibroblast cells, because they are abundantly present in many organs, including the skin [**55,56**], and can be obtained by non-invasive procedures. However, fibroblasts and ECs are epigenetically different, and express different sets of genetic markers that localize to different cellular compartments. Mature ECs are non-proliferative [**19–22**], but can proliferate in response to growth factor stimulation [**19–22,57,58**]. Similarly, fibroblasts can proliferate upon activation [**59–61**]. Interestingly, one recent study has described the ability of fibroblast cells to acquire EC morphology and incorporate into newly formed blood vessels in the aftermath of injury [**62**]. Therefore, we tested the hypothesis that by partially erasing the “epigenetic memory” of fibroblasts cells using chromatin-modifying agents, it might be possible to reprogram these cells into blood vessel forming cells. By subsequently adding a differentiation agent (specifically a GSK-3β inhibitor) to these cells, we converted them into OCT4+ and VEGFR-2/FLK1+ cells that formed tube-like structures in a two-dimensional (2D) matrix.

## Materials and methods

### Reagents and antibodies

TSA (cat#T1952), Aza (cat#A3656), 1× Dulbecco’s minimum essential media (DMEM), sterile 1× Phosphate Buffered Saline (PBS) (pH 7.4), 1× Tris Buffered Saline (TBS) (pH 7.4), Fetal Bovine Serum (FBS), Trizol, and dimethyl sulfoxide (DMSO) were purchased from InVitrogen (Carlsbad, CA). TDG (also called NP-12; cat#SML0339), 1000× protease inhibitor cocktail, glycine, methanol, sodium dodecyl sulfate (SDS), Ponceau-S, phalloidin–tetramethylrhodamine B isothiocyanate (PHD-TRITC) dye, and Matrigel were purchased from Sigma-Aldrich Chemicals Company (St. Louis, MO). Anti-human LPP3 antibody (cat#PA1-12680), ProLong Gold Antifade Mounting reagent with DAPI, and cell dissociation solution (0.25% trypsin-ethylenediaminetetraacetic acid [EDTA]) were purchased from Gibco-BRL/Invitrogen (Carlsbad, CA). Rabbit anti-human OCT3/4 polyclonal antibody (cat#GTX59610) was purchased from GeneTex Inc., (Irvine, CA). Rabbit anti-human VEGFR-2 (also known as FLK1; mAb#2479), anti-CD31 (mAb#3528), anti-Brachyury (mAb#81694), anti-N-cadherin (mAb #14215), and anti-Tubulin polyclonal (cat#2148) antibodies were purchased from Cell Signaling Technology (Danvers, MA). Anti-TIE-2 antibody (Ab#135671) was purchased from Abcam (Cambridge, MA). Acrylamide/bis-acrylamide, tetramethylethylenediamine (TEMED), ammonium persulfate, and secondary antibodies (goat anti-rabbit horseradish peroxidase [HRP] and goat anti-mouse HRP) were purchased from Bio-Rad Laboratories (Hercules, CA). All other biochemicals and solvents used in this study were analytical or molecular biology grade.

### Cells and cell culture

The methods used for cell culture have been previously described [**63–66**]. Primary normal, human adult dermal fibroblasts cells (hADFCs) (PCS-201-012™) at passage 1 were obtained from American Type Culture Collection (ATCC; Manassas, VA). hADFCs were cultured in 1× DMEM supplemented with 10% FBS, 1× streptomycin and penicillin antibiotic solution, 4 mM glucose, and 2.5 mM L-glutamine. Confluent hADFCs were passaged at a 1:4 ratio every 3 days using cell dissociation solution.

### Treatment of cells with epigenetic modifier

Stock solutions of Aza, a DNMT inhibitor, was dissolved at 1:1 ratio of acetic acid and water; TSA, an HDAC inhibitor, and TDG, a GSK-3β inhibitor, were prepared in sterile DMSO and stored in aliquots at -20°C. Growing hADFCs at passage 3 (p3) were detached using cell dissociation solution. Cells were re-suspended in complete DMEM media with FBS to inactivate the trypsin/EDTA. The number of cells was counted using a hemocytometer, and equal numbers of cells re-plated into eight 10 cm dishes containing 10 mL complete DMEM media. The final working concentrations of Aza, TSA, and TDG were prepared prior to use at optimized concentrations of 50 nM, 25 nM, and 60 nM, respectively. Experiments were repeated four times unless otherwise indicated, with triplicate samples in each experiment.

### Extraction of messenger RNA (mRNA) and quantitative reverse transcription (qRT)-polymerase chain reaction (PCR) assay

Extraction of mRNA, cDNA preparation, and qRT-PCR methods have been previously described [**63–66**]. Total mRNA was prepared by washing control hADFCs and those treated with Aza + TSA or Aza + TSA + TDG once with 1× cold PBS, then solubilizing cells in Trizol. The mRNAs were quantified by reading the optical density at a 260 nm wavelength using a spectrophotometer. Gene-specific primers were synthesized and supplied by IDT-DNA (Coralville, IA): *OCT4*: For-5'-GGAGGGAAGGTGAAGTTCAATG-3' and Rev-5'-CTATCTACTGTGTCCCAGGCTTCT-3'; *VEGFR-2*/*FLK1*: For-5'-GTGTGGTGTCAAAGTTTCAGGAAG-3' and Rev-5'-GGTTTACTGGAGTAGAGGCCAAAT-3'; LPP3: For-5’-AGTTCACCTTGATCATGATGGC-3’ and Rev-5’-ACACGAAGAAAACTATGCAGCA-3’; *18S rRNA*: For-5'-GGAGGTTCGAAGACGATCAGA-3' and Rev-5'-GCATCGTTTATGGTCGGAACT-3'; and Table 1.

**Table 1:** Human gene specific primers used for q-RT-PCR data shown in S1 Fig.

Gene	Forward (5'-3')	Reverse (5'-3')
*Nanog*	*CCTGAAGACGTGTGAAGATGAG*	*CCAGTGTCCAGACTGAAATTGA*
*CMYC*	*TTCTCTCCGTCCTCGGATTCT*	*CATCTTCTTGTTCCTCAGAGT*
*Sox2*	*AGTACTGGCGAACCATCTCTGT*	*AATTACCAACGGTGTCAACCTG*
*TERT*	*TGACACCTCACCTCACCCAC*	*CACTGTCTTCCGCAAGTT*
*Brachyury*	*AAGGACAAGGAAGTGAAAGCTG*	*GCTCCACTTCTCTCTCTGGTGT*
*FOXC1*	*CTCACCTCGTGGTACCTGAA*	*ACCGACTGGAAGTTCTGCTG*
*FOXC2*	*GAGAAGAAGGTGGTGATCAAGAG*	*AGCGTCTCCACCTTGGTGAT*
*VEGF-A*	*AAGAAATCCCGGTATAAGTCCT*	*ACAAATGCTTTCTCCGCTCTG*
*VE-cadherin*	*TTGGAACCAGATGCACATTGAT*	*TCTTGCGACTCACGCTTGAC*
*vWF*	*AGACCCAGTGCTGTGATGAGTA*	*GTTGTGGTACAGCCACAGTCAT*
*CD31*	*AGCCCTAGAAGCCAATTAGTCC*	*GCAATTCTTAGGGGACAGTGAC*
*Tie-2*	*GCTTGGACCCTTAGTGACATTC*	*CCAGGAGTGTGTAATGTTGGAA*
*EFNB-2*	*TATGCAGAACTGCGATTTCCAA *	*TGGGTATAGTACCAGTCCTTGTC *
*NRP2*	*GATTGTGTTCGAGGGAGTGATAG*	*AGTTCTCCAGTGGGACATCAGT*
*eNOS*	*GTTTGTCTGCGGCGATGTTAC*	*AATGTCTTCGTGGTAGCGTTG*
GAPDH	*TGTGGGCATCAATGGATTTGG*	*ACACCATGTATTCCGGGTCAAT*
*18s rRNA*	*GTAACCCGTTGAACCCCATT*	*CCATCCAATCGGTAGTAGCG *

### Biochemical methods

The immunoblotting methods have been previously described [**63–66**]. In brief, cell extraction buffer was prepared by mixing 25 mM Tris pH 7.4, 0.1% Triton X-100, 0.25% NP-40 detergent, 150 mM Sodium Chloride, 1 mM EDTA, and 25 mM Sodium Fluoride, 1 mM Sodium Orthovanadate, and 10 mM Sodium Pyrophosphate pH 7.5. The proteins were separated using a glass plate SDS-PAGE apparatus, then transferred to a nitrocellulose membrane. The membrane was incubated with the appropriate antibodies, washed with 1× TBST, then reacted with highly sensitive chemiluminescent (ECL) detection reagent (Thermo-Fisher, Rockford, IL). X-ray films were exposed to the nitrocellulose membrane and developed using an automated Kodak X-ray film developer.

### Cell staining and microscopy

The microscopy methods have been previously described [**63–66**]. In brief, the hADFCs were plated on sterile coverslips, incubated in media without additional compounds; with TSA and Aza; or with TSA, Aza, and TDG. The cells were fixed with 4% paraformaldehyde (PFA), washed three times with 1× TBS pH 7.4, permeabilized with 0.5% Triton X-100 in 1× TBS, and washed three times with 1× TBS. To visualize the fibroblastic phenotype of cells, hADFCs were stained with PHD-TRITC dye. Microscopic images were captured using an Olympus BX51 microscope with an Olympus 12.5MP DP71 CCD camera using an UPlanFL N dry 20×/0.75NA objective at room temperature, and images saved as TIFF files.

### Chromatin immunoprecipitation (ChIP) experiment

The ChIP experiments were carried out as previously described [**64,66**]. The Magna ChIP kit was purchased from Thermo-Fisher Scientific (Rockford, IL). The chromatin samples were pre-cleared, then subjected to immunoprecipitation with anti-human OCT4 antibody bound to beads. The chromatin immunoprecipitate complexes were analyzed by PCR using primer pairs (For-5'-*GGGAAATAGCGGGAATGTTG*-3' and Rev-5'-*GCGAAATGCCCAGAACTC*-3'; NCBI reference sequence: NC_000004.12) that amplify an ~850-bp DNA sequence in the region of the human *VEGFR-2/FLK1*-promoter/enhancer. The PCR product was resolved by 4% agarose gel in 1× TAE buffer, thereafter visualized by staining with 0.2 μg/ml ethidium bromide (EtBr), and images captured using a Polaroid gel documentation system.

### Tube formation in 2D Matrigel, paraffin embedding, sectioning and staining, microscopy

These methods have been previously described [**63,65–69**]. In brief, 5 × 10^5^ cells were plated in 24-well dishes. After 24 hours, the formation of capillary-like structures was examined under a light microscope at room temperature. Microscopic images were recorded by an Olympus BX51 microscope with an Olympus 12.5MP DP71 CCD camera using an UPlanFL N dry 20×/0.75NA objective.

### Statistics

Data obtained from at least three independent experiments (n = 3) are expressed as means ± standard errors of the mean (S.E.M.). Comparisons between two means were performed with an unpaired Student’s t-test. The differences among groups were evaluated by analysis of variance (ANOVA). P<0.05 was considered statistically significant.

## Results

### Gene expression profile of hADFCs

**Fig 1:**
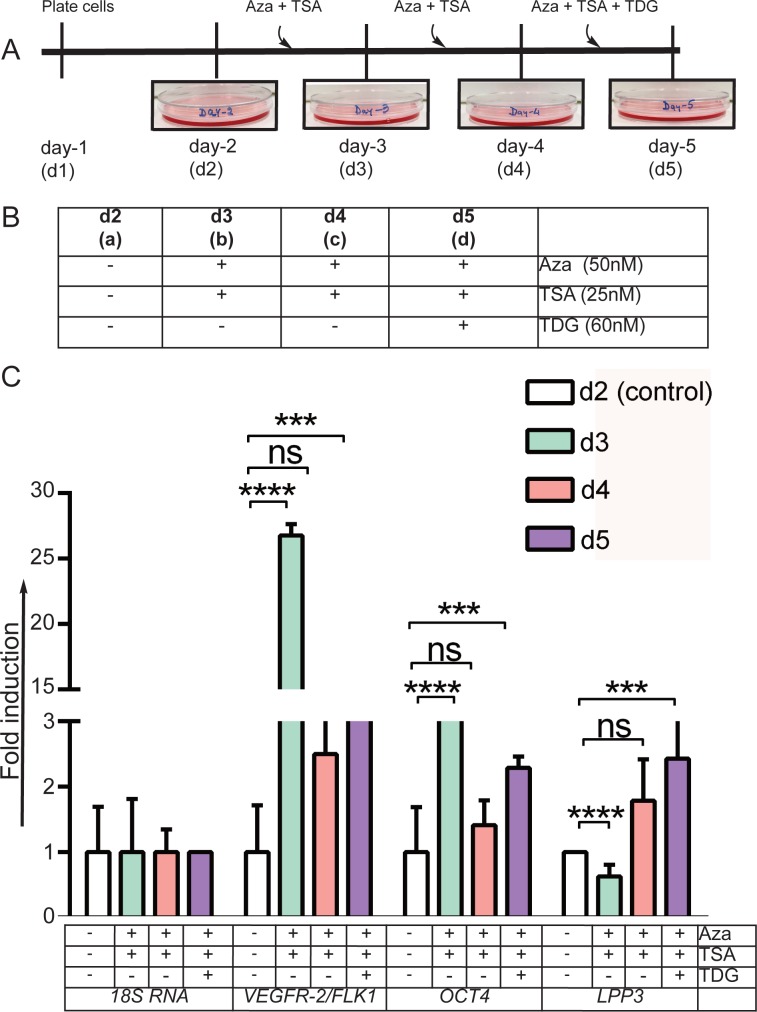
Chromatin modification strategy, timeline of experiments, and gene expression analyses. **A**) The strategy and timeline used for all the experiments described in this study. **B**) Descriptions of the four experimental groups: (**a**) day-2 (d2) dish is control untreated cells; (**b**) day-3 (d3) dish received one dose of Aza + TSA for 24 hours before the cells were used; (**c**) day-4 (d4) received a second dose of Aza + TSA for 24 hours before use; (d) day-5 (d5) received a third dose of Aza + TSA and TDG 24 hours before use. **C**) Total mRNAs prepared from these cells were subjected to q-RT-PCR using gene-specific primers for *18S RNA* (control), *VEGFR-2/FLK1*, *OCT4*, and *LPP3*. Experiments were carried out three times, with triplicate mRNAs. * p<0.05 or ** p<0.01 vs. control *18S RNA* from same group.

In pilot experiments, we optimized the doses of epigenetic modifiers Aza (50 nM) and TSA (25 nM), and differentiation factor TDG (60 nM) in hADFCs. At these concentrations, hADFCs were highly proliferative and the compounds were non-toxic (S1 Fig). We also optimized specific time points for this study (Fig 1A). The dose and the experimental groups are listed in Fig 1B. The experimental groups included: a) day-2 (d2) control, untreated; b) day-3 (d3), received one dose of Aza + TSA; c) day-4 (d4), received second dose of Aza + TSA; and d) day-5 (d5), received third dose of Aza + TSA as well as TDG. All cells were grown in complete media, not deprived of growth factor or serum. Initial pilot experiments were conducted to test if the exposure of hADFCs to chromatin modifying agents induced expression of pluripotency, mesodermal, and endothelial cell genes. Q-RT-PCR data showed expression of these genes fluctuated over time (S2 Fig). Specifically, we focused on *OCT4*, *VEGFR-2/FLK1*, and *LPP3* genes.

Next, to test the hypothesis that epigenetic modification might be adequate to reprogram fibroblast cells to blood vessel forming endothelial-like cells, we performed qRT-PCR to assay to examine the expression of pluripotency gene *OCT4*, and EC genes *VEGFR-2/FLK1* and *LPP3*. Control (d2) cells showed insignificant levels of *OCT4*, *VEGFR-2/FLK1*, and *LPP3*, whereas expression of these gene increased significantly in d3 and d4 (Aza + TSA), and d5 (Aza + TSA + TDG) cells (Fig 1C). Importantly, expression of the EC genes was not detectable in d2 cells, but increased significantly in d3, d4, and d5 cells (Fig 1C). However, expression of the *18S* RNA control remained unchanged in all conditions (Fig 1C). Although *Sox2*, *Nanog* and *KLF4* were detectable by q-RT-PCR (S2 Fig), their protein products were not detectable (S3 Fig). These data show that chromatin modification can promote activation of *OCT4*, *VEGFR-2/FLK1* and *LPP3* genes expression in these cells.

### Chromatin modification mediate the expression of β-catenin, OCT4, VEGFR-2/FLK1, Brachyury, N-cadherin, TIE-2, CD31, and LPP3 proteins in hADFCs

**Fig 2:**
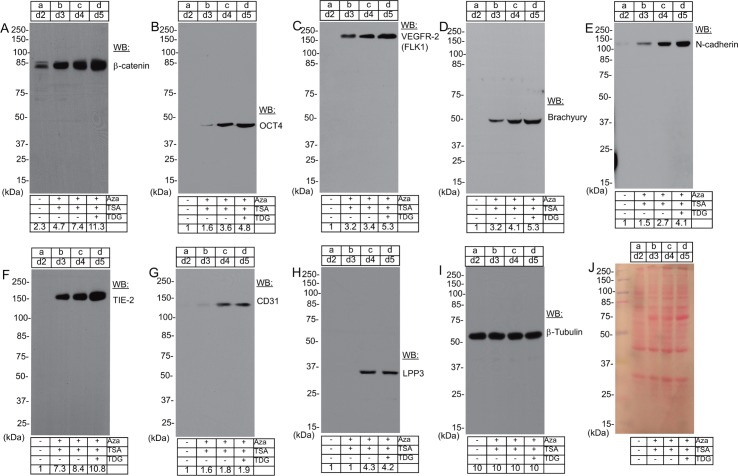
Chromatin modification mediate expression of mesodermal and endothelial cells marker proteins. Total protein extracts prepared from: a) d2 control cells; b) d3 cells treated once with Aza + TSA; c) d4 cells treated twice with Aza + TSA; d) d5, treated with a third dose of Aza + TSA and TDG were resolved by denaturing SDS-PAGE. Proteins were transferred onto a nitrocellulose membrane and analyzed by Western blotting (WB) with antibodies to: **A**) β-catenin, **B**) OCT4, and **C**) VEGFR-2/FLK1; **D**) Brachyury; **E**) N-cadherin; **F**) TIE-2; **G**) CD31; **H**) LPP3; and (**I**) β-tubulin. β-tubulin antibody was used to judge equal loading of proteins across the lanes. (**J**) Representative image of ponceau-S stained nitrocellulose membrane. Molecular weights are given in kiloDaltons (kDa). The numbers at the lowermost rows of each image indicate signal intensities that were quantified by NIH ImageJ. Experiments were carried out three times.

To address the hypothesis that epigenetic modification might be sufficient to convert hADFCs to immature or endothelial-like cells, we performed Western blot analysis. Cell extracts prepared from these cells showed basal levels of β-catenin in d2 control cells (Fig 2A). However, the level of β-catenin increased in d3, d4, and d5 cells (Fig 2A). We observed a very high level of β-catenin at d5 cells. Because of the inhibition of GSK-3β, d5 cell extract also likely contains non-phosphorylated (i.e., stabilized) β-catenin protein, so the increase in levels may indicate enhanced expression, stabilization, or both. TDG alone did not increase the level of β-catenin polypeptide species in control hADFCs (S4 Fig). In d2 control cells, OCT4, VEGFR-2/FLK1, Brachyury, N-cadherin, TIE-2, CD31, and LPP3 proteins were not detectable (Fig 2B–2H). However, we observed significant increases in OCT4, VEGFR-2/FLK1, Brachyury, N-cadherin, TIE-2, CD31, and LPP3 proteins in d3 and d4 cells, and the highest levels were observed in d5 cells (Fig 2B–2H). To determine if VEGFR-2/FLK1 protein was localized at the cell surface, these cells were subjected to cell surface labeling with anti-VEGFR-2/FLK1 antibody and analyzed by Fluorescence activated cell sorting (FACS) assay (S5 Fig). As expected, cell surface expression of VEGFR-2/FLK1 was increased steadily from d3-d5, while isotype matched control did not (S5A–S5D Fig). We also detected expression of mesodermal transcription factor Brachyury (Fig 2D). Although VE-cadherin was undetectable (S6 Fig), on d4 and d5 the modified cells expressed N-cadherin (Fig 2E). Moreover, the expression of endothelial cell markers TIE-2 (Fig 2F), CD31 (Fig 2G), and LPP3 (Fig 2H) were increased. In contrast, Western blot analyses failed to detect the expression of Nanog, KLF4 and Sox2 proteins in these cell lysates (S3 Fig). We used an anti-tubulin (Fig 2I) antibody as well as Ponceau-S staining (Fig 2J) of the nitrocellulose membrane to confirm equal loading of proteins across the lanes. These data show that chromatin modification can mediate expression of β-catenin, OCT4, VEGFR-2/FLK-1, Brachyury, N-cadherin, TIE-2, CD31 and LPP3 proteins in hADFCs.

### In epigenetically modified fibroblasts β-catenin mostly localizes to the cell-cell junctions, while OCT4 localizes to the nucleus

**Fig 3:**
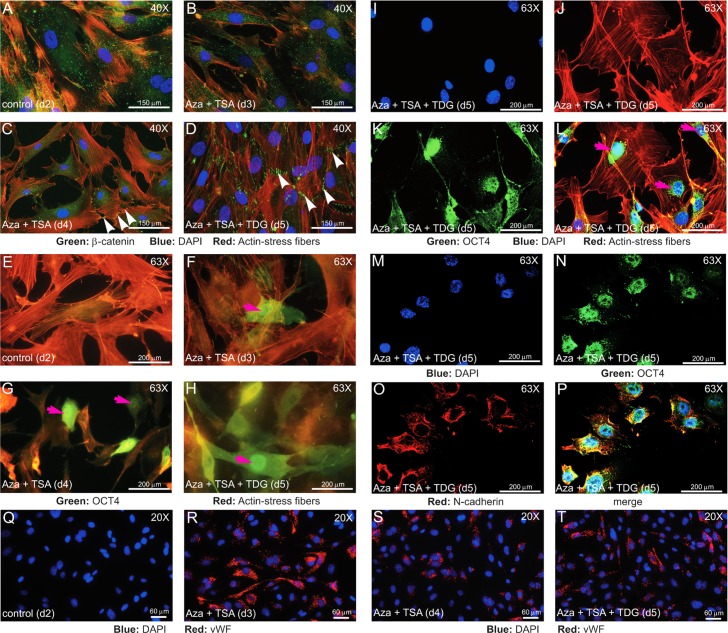
Microscopic analysis of β-catenin, OCT4, N-cadherin and vWF in epigenetically modified cells. hADFCs were plated on coverslips, left untreated or treated with epigenetic modifiers as described in Fig 1A, and stained with anti-β-catenin (A-D, green) or anti-OCT4 (E-P, green) or anti-vWF (Q-T, red). Representative microscopic images of: **A&E**) d2 control untreated cells; **B&F**) d3 cells treated once with Aza + TSA; **C&G**) d4 cells received two doses of Aza + TSA; **D&H**) d5, treated with a third dose of Aza + TSA and TDG. White arrowheads indicate β-catenin; magenta arrowheads show nuclear accumulation of OCT4. **I-L**) d5 cells treated with a third dose of Aza + TSA and TDG were stained with DAPI (blue), TRITC-phalloidin (red), and OCT4 (green). Magenta arrows (**L**) indicate nuclear localization. **M-P**) d5 cells treated with a third dose of Aza + TSA and TDG were stained with DAPI (blue), OCT4 (green), and N-cadherin (red). **Q-T**) d2, d3, d4 and d5 hADFC were stained with DAPI (blue) and vWF (red). Magnification is as shown.

Next, we used epifluorescence microscopic imaging to examine the distribution of β-catenin, OCT4, N-cadherin, and von Willebrand factor (vWF) in control hADFCs and those treated with Aza and TSA with or without TDG as described in Fig 1A. β-catenin was diffusedly distributed in d2 and d3 cells, while its localization increased in cell-cell junctions in d4 and d5 cells (Fig 3A–3D). We observed OCT4 staining primarily in the nucleus (Fig 3E and 3F, green), with some localization in the cytoplasm in d3, d4, and d5 cells (Fig 3E and 3F, green, arrows). Co-staining of d5 cells with DAPI showed increased OCT4 in the nucleus (Fig 3I–3L, magenta arrows). D5 cells had increased OCT4 compared to d4 cells. These cells were also stained with phalloidin-TRITC (red) to visualize actin cytoskeleton (Fig 3A–3L). In these cells, VE-cadherin was not detectable (S7 Fig), while N-cadherin was diffusedly distributed throughout the cells and OCT4 in the nucleus (Fig 3M–3P and S8 Fig). The normal staining pattern and the presence of actin stress fibers indicated that these cells appeared to be adherent phenotype, rather than anchorage-independent cancer cells. Importantly, d3, d4 and d5 cells expressed vWF (Fig 3Q–3T). Together, these data confirm that OCT4 was expressed in epigenetically modified fibroblast cells and reveal that it does translocate to the nucleus. In addition, at d3, d4, and d5 cells also expressed endothelial markers N-cadherin, TIE-2, CD31, LPP3 and vWF proteins (Fig 2).

### OCT4 binds to the *VEGFR-2/FLK1* promoter/enhancer region and transcriptionally activates the expression of *VEGFR-2/FLK1* in epigenetically modified cells

**Fig 4:**
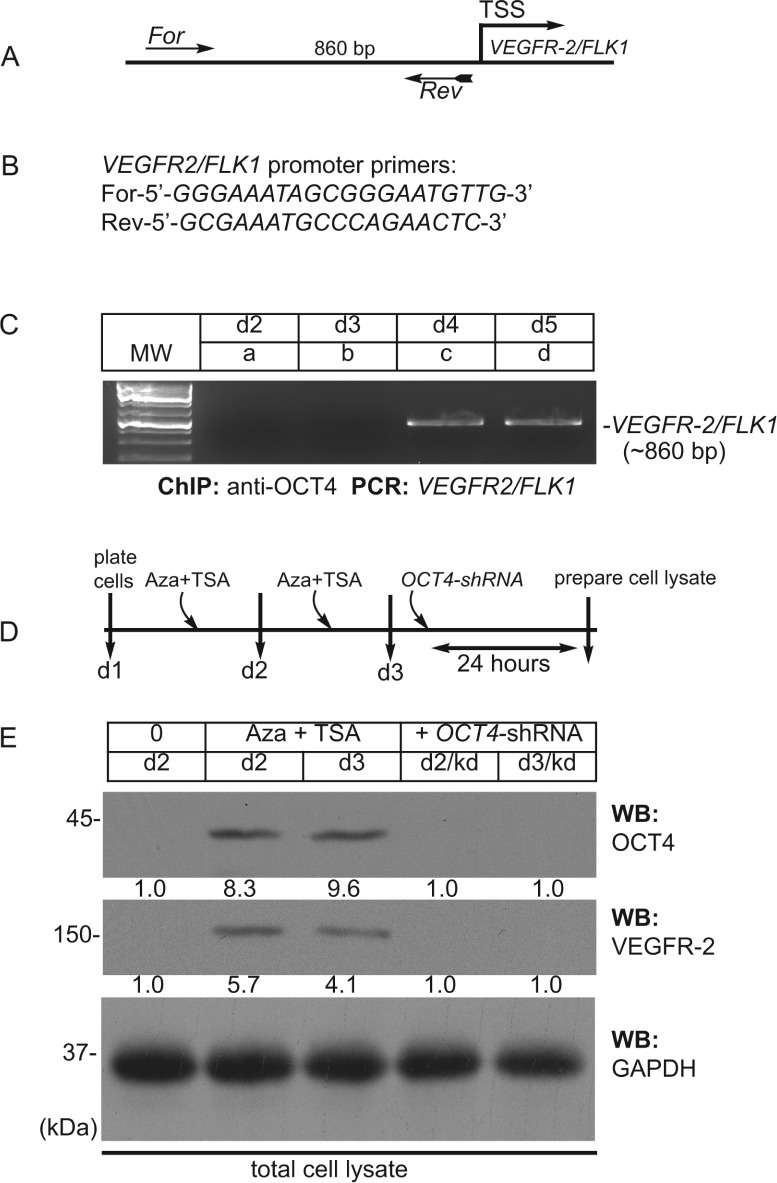
In epigenetically modified hADFCs OCT4 binds to the *VEGFR-2/FLK1*-promoter sequence. **A)** Schematic of the human *VEGFR-2/FLK1*-promoter region. The approximate location of the forward (For) and reverse (Rev) primers used to amplify the ~860-bp PCR product upstream of the transcription start site (TSS) is shown. **B**) Primers used for amplifying OCT4 binding region in *VEGFR2/FLK1* promoter. **C**) Representative ethidium bromide (EtBr) stained agarose gel showing PCR product obtained from anti-OCT4 ChIP experiment using chromatin from **a**) d2 control untreated cells; **b**) d3 cells treated once with Aza + TSA; **c**) d4 cells treated twice with Aza + TSA; **d**) d5, treated with a third dose of Aza + TSA and TDG. **D**) Strategy, timeline of Aza + TSA treatment and OCT4-knockdown. **E**) Total cell extracts prepared from: d2 control hADFCs; d2 cells receiving Aza and TSA; d3 cells receiving second dose of Aza and TSA; d2/kd, d2 cells receiving Aza and TSA were subjected to *OCT4*-shRNA-knockdown (d2/kd); d3 cells receiving second dose of Aza and TSA were subjected to *OCT4*-shRNA-knockdown (d3/kd) were subjected to WB with indicated antibodies. GAPDH was used to judge equal loading of proteins across the lanes. The numbers below the OCT4 and VEGFR-2 WB panels represent WB signal intensities. Experiments were repeated three times.

Because we found high levels of OCT4 expression in epigenetically modified cells (Figs 1 and 2), and OCT4 regulates transcription of critical pluripotency/stemness genes, we examined if OCT4 regulated gene expression in these hADFCs. Accordingly, we performed the ChIP assay to identify sites bound by OCT4 in the *VEGFR-2/FLK1*-promoter/enhancer DNA region (Fig 4A and S9 Fig). DNA sequence analysis identified five putative OCT4-binding sites (G/CA/TNATGC) within the 860-bp genomic segment upstream of the *VEGFR-2/*FLK1 transcription start site (Fig 4B). Using the ChIP assay, we did not observe any binding of OCT4 to the *VEGFR-2/FLK1* promoter/enhancer DNA segment in d2 (control) or d3 cells (Fig 4C, lanes a and b). In contrast, OCT4 bound to the *VEGFR-2/FLK1* promoter/enhancer DNA segment in d4 and d5 cells (Fig 4C, lanes c and d), while OCT4 did not bind to the *LPP3* promoter/enhancer (S10 Fig). To address the relationship of OCT4 binding to the *VEGFR-2/FLK1-*promoter/enhancer DNA segments, we knocked-down *OCT4* using *OCT4-shRNA* and proteins extracts were analyzed by Western blotting (Fig 4D and 4E). The timeline of Aza + TSA treatment and *OCT4-shRNA*-mediated knockdown in hADFCs is shown (Fig 4D). Accordingly, *OCT4*-knockdown of d2 or d3 hADFCs decreased the expression of OCT4 and VEGFR-2/FLK1 proteins. These data show that OCT4 binds to and transcriptionally activates the expression of the *VEGFR-2/FLK1* gene.

### Epigenetically modified fibroblast cells form capillary tube-like structures

**Fig 5:**
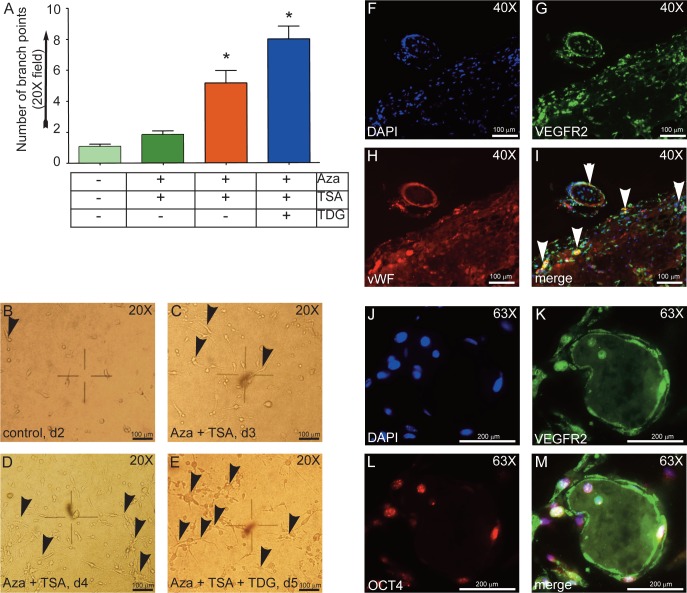
Epigenetically modified hADFCs form capillary tube-like structures in 2D Matrigel tube assay. 5 × 10^5^ cells were plated on 2D Matrigel for 24 hours, then examined under phase contrast microscope at the indicated magnification. **A**) Quantification of branch point structures. Experiments were conducted three times. Data represent means ± SEM. *p<0.05 vs. control untreated group. **B-E**) Representative images of branch point structures. Black arrowheads indicate branch points, where two or more cells make cell-cell connections. Representative images of paraffin embedded Matrigel section were stained with: **F-I**) DAPI (blue, nucleus), anti-VEGFR-2/FLK1 (green), and anti-vWF (red) antibodies. **J-M**) DAPI (blue, nucleus), anti-VEGFR-2/FLK1 (green), and anti-OCT4 (red). Magnification is as shown.

Next, we subjected hADFCs onto 2D Matrigel assay and examined whether the epigenetically modified hADFCs behaved as ECs. Only ECs are known to form branching capillary tube-like structures in 2D gel [**66,68**]. The Matrigel assay is considered an *in vitro* correlate of angiogenesis [**66,68**]. Thus, to assess the potential of our epigenetically modified cells to form branch point structures *in vitro*, we plated these cells onto growth factor reduced 2D Matrigel and quantified the formation of branch points (Fig 5A). Plating of control fibroblast cells (d2) did not result in any network-like arrangements (Fig 5A and 5B). In contrast, d3, d4, and d5 cells formed branching, network-like structures. Arrowheads indicate branch points (Fig 5B–5E). To characterize and identify the tube forming cells, d5 Matrigels were embedded in paraffin, sectioned and stained either with VEGFR-2/FLK1 and vWF (Fig 5F–5I), or VEGFR-2/FLK1 with OCT4 (Fig 5J–5M). We show that d5 cells in 2D Matrigel not only formed branching points, but also retained expression of OCT4 and VEGFR-2/FLK1 proteins. In the absence of VEGF, however, these cells did not form branch point structures (S11 Fig). Thus, epigenetically modified fibroblasts cells transitioned into endothelial-like cells and the increase in VEGF receptor expression allowed the cells to respond to VEGF to form capillary-like structures.

## Discussion

**Fig 6:**
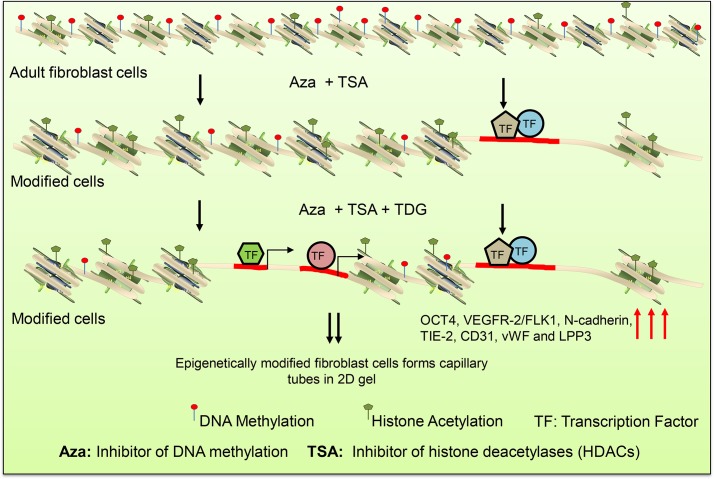
A model of the conversion process of fibroblast cells into tube-forming cells. hADFCs are mature differentiated cells with highly condensed chromatin containing histones tightly bound to DNAs. However, upon treatment with Aza and TSA for two or three cycles, histone acetylation increases, DNA methylation decreases, the chromatin becomes relaxed, so that the transcription factors could activate the expression of mesodermal-endothelial genes. These events mediate the formation of tube-like structures in 2D Matrigel in a VEGF-dependent manner.

In this study, we showed that the hADFCs treated with chromatin modifying agents, thereafter with TDG mediated: (a) higher expression of *OCT4*, *VEGFR-2/FLK1* and *LPP3* mRNAs; (b) increased levels of β-catenin, OCT4, VEGFR-2/FLK1, Brachyury, N-cadherin, TIE-2, CD31 and LPP3 proteins; (c) mediated accumulation of OCT4 protein in the nucleus; (d) OCT4 protein binding to the *VEGFR-2/FLK1* promoter/enhancer; and (e) formation of capillary tube-like vascular structures in 2D Matrigel in a VEGF-dependent manner (Fig 6).

We treated the fibroblast cells with optimized doses of Aza and TSA for 3 days. If the cells were treated with these chemical compounds only once over 2 days, there was no significant induction of OCT4. However, if the cells were allowed to grow in the presence of these two compounds (Aza and TSA) and let these cells cycle for at least one round, before an additional stimulation with Aza and TSA, the cells expressed higher levels of β-catenin and OCT4. Importantly, if we let these cells cycle for two to three rounds (equivalent to d4 and d5 cells), the expression of OCT4, VEGFR-2/FLK1, Brachyury, N-cadherin, TIE-2, CD31 and LPP3 increased significantly. These results suggest that for effective epigenetic modification to occur, the cells must be cycling actively at least for 3 to 4 rounds. Thus, we followed the same experimental strategy and timeline (Fig 1A) throughout this study. In pilot experiments, we observed expression of *OCT4*, *Sox2*, *Nanog*, and *Klf4* genes, however, their corresponding protein products were not detectable by Western blot. Although, the level of expression of OCT4 was increased at d4 and d5, these cells did not acquire a pluripotent stem cell state. In other words, the epigenetic change that occurred in these cells were incomplete, heterogeneous, or both. One could surmise that for these cells to become pluripotent stem cells there could be a threshold level of expression of OCT4 that is required to convert fully these cells to pluripotent stem cell state. Alternatively, there might be other unknown mechanisms that prevent OCT4 from exerting its pluripotency function, e.g., by associating with a transcription repressor complex [**70–73**].

Fibroblasts are abundant in many organs throughout the body [**74–76**]. For instance, skin is a very good source of fibroblast cells [**76**]. Fibroblast cells can also be harvested from blood cells [**76**]. Literature show that human umbilical cords, which are often discarded in the operating rooms, can be used as a source of neonatal fibroblast cells. Fibroblast cells do not usually express key stem cell TFs such as *OCT4* or *Nanog*. OCT4 expression is only found in stem cells and iPS cells. Accordingly, control hADFCs did not express *OCT4* mRNA or protein; however, *OCT4* and its effector *VEGFR-2/FLK1* were expressed at very high levels in hADFCs treated twice with Aza + TSA as well as those treated additionally with all three compounds. These data indicate the ability of TSA and Aza to mediate the activation of *OCT4* and *VEGFR-2/FLK1* genes. Because OCT4 is a stem cell TF, it likely binds to many promoter/enhancer sites to activate various genes. We also observed increased expression of *Sox2*, *Nanog* and *KLF4* genes, but their corresponding protein products were not detectable by Western blot (S3 Fig). If not pluripotency, what is the significance of increased OCT4 expression? One could surmise that there might be more than one pool of OCT4 protein complexes in the nucleus, and some OCT4-protein could be sequestered by the transcriptional repressor proteins. Thus, the level of OCT4 protein that is transcriptionally competent in chromatin modified hADFCs may not be sufficiently high enough to exert pluripotency. We treated cells with TDG to activate Wnt signaling, because Wnt signaling is required for differentiation of ECs [**20,40,44**]. It appears that TDG induced the highest level of β-catenin accumulation and allowed sustained expression of *OCT4* and *VEGFR-2/FLK-1*. Increased β-catenin level is likely driven by several transcription factors including canonical T-cell factor (TCF)/lymphoid enhancer factor (LEF-1) transcriptional network. In this regard, we currently do not fully understand the role of TDG. To examine whether these cells can transition into vascular ECs, we chose to focus on the VEGFR-2/FLK-1 receptor protein. Genetic studies have shown that without *VEGFR-2/FLK-1*, mouse embryos die *in utero* due to a lack of vascular ECs [**40**]. Accordingly, the binding of VEGF to VEGFR-2/FLK1 modulates several aspects of blood vessel formation, including EC development, migration, proliferation, survival, differentiation, and tube formation [**20,40,42]**. Thus, our data indicate that epigenetic modification of fibroblasts can lead to the formation of OCT4+ and VEGFR-2+ cells *in vitro*.

We examined levels of VEGFR-2/FLK1 to address whether fibroblast cells can acquire a mesodermal phenotype, because VEGFR-2/FLK1+ mesodermal cells are known to give rise to ECs, smooth muscle cells, cardiomyocytes, and hematopoietic stem cells. VEGFR-2/FLK1 expression, which was sustained in our modified hADFCs, is necessary for EC identity and required to enable ECs to form blood vessels during development and wound healing [**20,40,42**]. VEGFR-2/FLK1 is considered a *bona fide* marker for mesodermal cells, ECs, and cardiomyocyte cells. We acknowledge that in epigenetically modified fibroblast cells, VEGFR-2/FLK1+ cells may include several types of mesodermal precursor cells rather than ECs alone. However, staining for β-catenin demonstrated the ability of these cells to form cell-cell junctions, much like ECs. These results indicate that we were able to partially remove “epigenetic memory” in these cells, which resulted in the expression of OCT4, VEGFR-2/FLK1, and LPP3. As these cells also expressed N-cadherin, CD31, TIE-2, LPP3 and vWF, and formed tube like-structures, we posit that epigenetic reprogramming might be one of the ways to produce vascular endothelial cells from fibroblasts. Complete reprogramming of somatic cells into iPS cells require a genome-wide “relaxed” chromatin state, characterized by Histone-H3 trimethyl Lysine-4 (H3K4me3) enrichment and decreased methylation epigenome. This event concurrently induces re-expression of *OCT4*, *SOX2*, and *KLF4* stemness genes, preceded by gene promoter demethylation. Currently it is unknown, if only a subset of hADFCs might be epigenetically modified. Nevertheless, our results are important and significant, as they show the potential of Aza, TSA, and TDG to reprogram fibroblast cells into immature cells without the use of viral vectors. Importantly, the capacity to reprogram a large number of functional ECs from fibroblast cells, and the potential of these ECs to re-vascularize ischemic cardiovascular organs, will be of significant value in regenerative medicine.

## Conclusion

Here, we showed that hADFCs can be epigenetically modified to express high levels of OCT4, VEGFR-2/FLK-1, N-cadherin, TIE-2, CD31, LPP3 and vWF proteins, and these cells formed capillary tube-like structures in 2D gel. Their ability to form vascular capillary-like structures highlights the potential of autologous modified endothelial-like cells to form new blood vessels, suggesting they might aid in neovascularization and rescuing ischemic cardiovascular tissues. However, further studies will be required to address if modified cells form blood vessels in clinically relevant animal experiments.

## Supporting information

S1 Fig Chromatin modifying agents induced hADFCs proliferation. **A**) Timeline of cell proliferation (BrdU) assay. hADFCs (2 x 10^5^) treated with Aza (50 nM), TSA (25 nM), or TDG (60 nM), and BrdU for indicated time periods. **B**) Quantification of BrdU incorporation in hADFCs in response to indicated modifying agents. Data represent means ± S.E.M. Experiments were carried out three times, with triplicate samples. * p <0.05 vs control.
(EPS)

S2 Fig Quantitative (q) Reverse Transcription (RT)-PCR analysis of gene expression in control d2 hADFC, and chromatin modified d3, d4, and d5 cells. Total mRNAs were subjected to q-RT-PCR experiment with indicated gene specific primers (Table 1). The expression of several mesodermal and endothelial cell specific genes at d3 and d4 appear to fluctuate.
(EPS)

S3 Fig Klf4, Nanog and Sox2 proteins are not detectable in control d2, and chromatin modified d3, d4, and d5 hADFCs. **A**) Timeline of hADFCs treatment with epigenetic modifiers. **B**) Equal amount of total proteins prepared from d2, d3, d4, d5, and control human U87 glioblastoma cell line (ctrl) were separated by SDS-PAGE and analyzed by Western blotting with indicated antibodies. Molecular weights are given in kiloDalton (kDa).
(EPS)

S4 Fig TDG alone does not alter the expression of β-catenin in hADFCs. **A**) Timeline of hADFCs treatment with TDG. **B**) Equal amount of total proteins prepared from d2, d3, d4, and d5 were resolved by SDS-PAGE and analyzed by Western blotting with indicated antibodies. Anti-β-tubulin was used to determine equal loading of proteins across the lanes. Molecular weights are given in kiloDalton (kDa).
(EPS)

S5 Fig Cell surface analysis of VEGFR-2/FLK1 protein. Indicated cells at d2, d3, d4 and d5 (2 x 10^5^) were detached non-enzymatically from culture dishes, neutralized by washing twice with 1x PBS, incubated with isotype matched control IgG (2.0μg/ml) or with anti-VEGFR-2/FLK1 antibody, thereafter incubated with donkey anti-mouse IgG conjugated to Fluorescein isothiocyanate (FITC).
(EPS)

S6 Fig VE-cadherin is not detectable in control d2, and chromatin modified d3, d4, and d5 hADFCs. **A**) Equal amount of total proteins prepared from d2, d3, d4, d5, and control human umbilical vein endothelial cells (HUVECs) were separated by SDS-PAGE, thereater analyzed by Western blotting with indicated antibodies. The membrane was intentionally overexposed to reveal minor non-specific signals present in d4 and d5 lanes. The fast moving anti-VE-cadherin antibody reactive polypeptide species are likely non-specific signals. **B**) The nitrocellulose membrane was stripped and reprobed with anti-GAPDH to estimate equal loading of proteins across the lanes. The Molecular weights are given in kiloDalton (kDa).
(EPS)

S7 Fig VE-cadherin is undetectable in chromatin modified hADFCs. Control HUVECs and indicated cells were plated on coverslips, left untreated or treated with epigenetic modifiers, as described in S3 Fig, and stained with anti-VE-cadherin. Representative microscopic images of control ECs, d2, d3, d4 and d5 cells stained with anti-VE-cadherin (green) and DAPI (blue). Magnification is as shown. Scale bar, 150 μm.
(EPS)

S8 Fig Localization of N-cadherin in epigenetically modified cells. hADFCs were plated on coverslips, left untreated or treated with epigenetic modifiers as described in Fig 1A and Fig 4, and stained with anti-N-cadherin antibody (green) and TRITC-phalloidin (red). Representative microscopic images of: **A)** d2 control untreated cells; **B**) d3 cells treated once with Aza + TSA; **C**) d4 cells treated twice with Aza + TSA; **D**) d5, treated with a third dose of Aza + TSA and TDG. Approximately 10–20% of N-cadherin appears to be in the membrane (green arrows), while this protein is mostly diffusedly distributed elsewhere. **E-H**) d5, receiving a third dose of Aza + TSA and TDG were stained with DAPI (blue), N-cadherin (green), OCT4 (red). OCT4 is found in the nucleus and in cytoplasm. Magnification is as shown.
(EPS)

S9 Fig Human VEGFR-2/FLK1 promoter DNA sequence. (PDF)

S10 Fig OCT4 does not bind to the human LPP3-promoter sequence. **A**) Human LPP3 promoter DNA sequence ~1100bp upstream of transcription start site (TSS). Shaded and underlined DNA sequences represent the primers. **B**) Schematic of promoter/enhancer region of the human LPP3 gene showing approximate locations of forward and reverse primers used for ChIP PCR. **C**) LPP3-promoter primer DNA sequences. **D**) Ethidium Bromide (EtBr) stained agarose gel shows no PCR amplification product.
(PDF)

S11 Fig Epigenetically modified hADFCs plated in 2D Matrigel fail to form tube-like structures in absence of VEGF. **A**) Timeline of epigenetic modification and 2D Matrigel assay. hADFCs were plated on Matrigel as described in Fig 5 and allowed to form tube-like structures. **B-E**) Representative images of chromatin modified hADFCs that failed to elongate, make cell-cell connections or form branching point structures in 2D Matrigel. Magnification is as sown. Scale bar, 50 μm.
(EPS)
