# Supplementary material for: Chromatin-modifying agents convert fibroblasts to OCT4+ and VEGFR-2+ capillary tube-forming cells
Source: PLoS One. 2017 May 3;12(5):e0176496. doi: 10.1371/journal.pone.0176496 (PMC5415225; doi:10.1371/journal.pone.0176496)
Supplement: S9 Fig — (PDF) [file pone.0176496.s009.pdf]

S9 Fig. Human *VEGFR2/FLK1* promoter DNA sequence.

1 AACCAGATTC AGCTTTTTAA ACTACAATTA TACTGGCCAA ACAAAATACC

51 CTTATACAAA AACCAAAACT ACTGGCAGGA GTCGCTGCCA GCTTGCGACC

101 CGGCATACTT GGCTGAGTAT CCGCTTCTCC CTTGTGGCTC CAAACTGCTG

151 CAGATTCTCG GCCACTTCAG ACGCGCGCGA TGGCGAAGAG GGTCCTGCAC

201 TTTGACGCGC CTGGTGAGGG AGCGCTGCTC TTCGCAGCGC TCCTGGTGAT

251 GCTCCCCAAA TTTCGGGGAC CGGCAAGCGA TTAAATCTTG GAGTTGCTCA

301 GCGCCCGTTA CCGAGTACTT TTTATTTACA CCAGAAACAA AGTTGTTGCT

351 CTGGGATGTT CTCTCCTGGG CGACTTGGGG CCCAGCGCAG TCCAGTTGTG

401 TGGGGAAATG GGGAGATGTA AATGGGCTTG GGGAGCTGGA GATCGCCGCC

451 GGGTACCCGG GTGAGGGGCG GGGCTGGCCG CACGGGAGAG CCCCTCCTCC

501 GCTCCGGCCC CGCCCCGCAT GGCCCCGCCT CCGCGCTCTA GAGTTTCGGC

551 ACCAGCTCCC ACCCTGCACT GAGTCCCGGG ACCCCGGGAG AGCGGTCAAT

601 GTGTGGTCGC TGCGTTTCCT CTGCCTGCGC CGGGCATCAC TTGCGCGCCG

651 CAGAAAGTCC GTCTGGCAGC CTGGATATCC TCTCCTACCG GCACCCGCAG

701 ACGCCCCTGC AGCCGCGGTC GGCGCCCGGG CTCCCTAGCC CTGTGCGCTC

751 AACTGTCCTG CGCTGCGGGG TGCCGCGAGT TCCACCTCCG CGCCTCCTTC

801 TCTAGACAGG CGCTGGGAGA AAGAACCGGC TCCCGAGTTC TGGGCATTTC

851 GCCCGGCTCG AGGTGCAGG**A TG**

Figure S2: ***Localization of N-cadherin in epigenetically modified cells.*** hADFCs were plated on coverslips, left untreated or treated with epigenetic modifiers as described in Figure 1A and Figure 4, and stained with anti-N-cadherin antibody (green) and TRITC-phalloidin (red). Representative microscopic images of: **a)** day-2 control untreated cells; **b**) day-3 cells treated once with Aza + TSA; **c**) day-4 cells treated twice with Aza + TSA; **d**) day-5, treated with a third dose of Aza + TSA and TDG. Approxiametly 10-20% of N-cadherin appear to be in the membrane (green arrows), while this protein is mostly diffusedly distributed elsewhere. Magnification is as shown
