# Supplementary material for: Chromatin-modifying agents convert fibroblasts to OCT4+ and VEGFR-2+ capillary tube-forming cells
Source: PLoS One. 2017 May 3;12(5):e0176496. doi: 10.1371/journal.pone.0176496 (PMC5415225; doi:10.1371/journal.pone.0176496)
Supplement: S10 Fig — A) Human LPP3 promoter DNA sequence ~1100bp upstream of transcription start site (TSS). Shaded and underlined DNA sequences represent the primers. B) Schematic of promoter/enhancer region of the human LPP3 gene showing approximate locations of forward and reverse primers used for ChIP PCR. C) LPP3-promoter primer DNA sequences. D) Ethidium Bromide (EtBr) stained agarose gel shows no PCR amplification product. (PDF) [file pone.0176496.s010.pdf]

S10 Fig. A) Human *LPP3* promoter/enhancer DNA sequence (primary source HGNC:HGNC:9229)

```
   1 GTTATCTCTA GTTACATACG GGGAAACTGA GGCACAGAGT CACAGGATTG AAATCCAGGA
  61 GATGTGGTTC CACAGTCGTG CAGTCTTTTT CATTTTAGTA TTCTCAAAAT CTAGCGTAGT
 121 ACCCGGTGTT GGATGTTAAG ATCTTAAACA AACAAACAAC TTGTTTTCCT TAAAAATTAT
 181 ATTTTCAGGG TAATACAATT CAAGTCACAA TAAAGACCTG AAAGCCTCAG GTTTGTAGGT
 241 GAGGAAAAAT GCATTTTAGC TGTACAGCCC TAACTTTTAC TTGACCCAAG GCCAGCAAGA
 301 AAATGTTTCT TTTTATTTAT ATATATTTTT TCTTTGGTGA CAATTTGGTG TTTCTGAACG
 361 CTAGGGGCTC CCGGTTTCTC TGGTTTGAGA GTAACTTTTC CTTTTAGGAC TTTTTTTTTT
 421 TTTTTTGAAG GGGGTGGGGG AAAATGTGGC CTTAATTATC CTACGTCTTA GGCAGCTTAA
 481 GGAAGGGGCT GTGCTTTCGG AATCCCATCC GAGCCAAGTA AGGAGGTCCC TCTCTCTCTC
 541 TCCCCCCACG TCTTCTCTTT TTAAAGGACC TCGTGAAATA AAAGTGCAGA AAACAAACCC
 601 AGGCGATCAC AGCAGCAGCC GCCGCGGCAG CAGCACCAAC AGCAGGAGGA GCAGGAGGAG
 661 CCGGAGGAGG AGGAGGAGGA GGAGGCAAAG TTAGAGTTGG GGCTGGCGCT CCGGAGTTGC
 721 TGGGCTCAGC GCAGCTCCCA TTCATTAAGG AACCAGCTGC GGAGGAAGGT GGCCGAGCGC
 781 CCGCGCTGCC CACTCGCTCG CTCGCGCACT CAGACGCGCG CCACAACAGC GCGCCCCAAG
 841 CTGCGCAGCT CTGCAAAAGT TTCTGCTCGG GATCTGGCTC TCTTCCCCTT GGACTTTAGA
 901 ACGATTTAGG GTTGACAGAG GAAAGCAGAG GCGCGCAGGA GGAGCAGAAA ACACCACCTT
 961 CTGCAGTTGG AGGCAGGCAG CCCCGGCTGC ACTCTAGCCG CCGCGCCCGG AGCCGGGGCC
1021 GACCCGCCAC TATCCGCAGC AGCCTCGGCC AGGAGGCGAC CCGGGCGCCT GGGTGTGTGG
1081 CTGCTGTTGC GGGACGTCTT CGCGGGGCGG GAGGCTCGCG CCGCAGCCAG CGCCATG
```

B

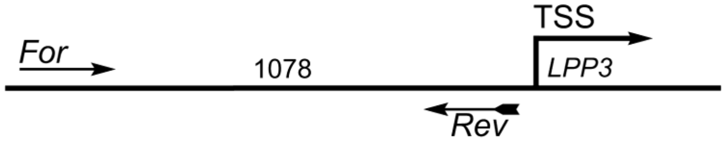

C

LPP3 promoter primers:
For: 5'-*CTGAGGCACAGAGTCACAGGATTG*-3'
Rev: 5'-*CGCGAAGACGTCCCGCAACAGCAG*-3'

D

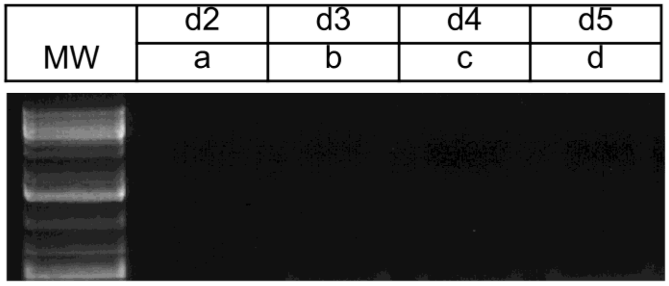

ChIP: anti-OCT4 **PCR:** *LPP3*
